# Supplementary material for: Type I interferon regulates interleukin-1beta and IL-18 production and secretion in human macrophages
Source: Life Sci Alliance. 2024 Mar 25;7(6):e202302399. doi: 10.26508/lsa.202302399 (PMC10963587; doi:10.26508/lsa.202302399)

Figure 4J WB source data

Supernatant anti-IL-1β

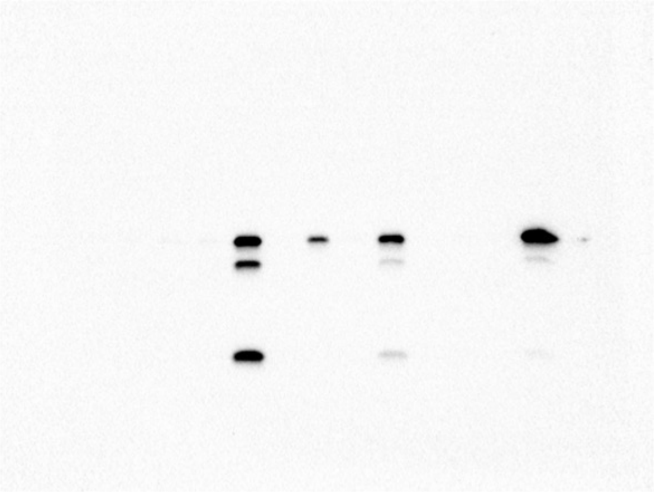

Lysate anti-IL-1β

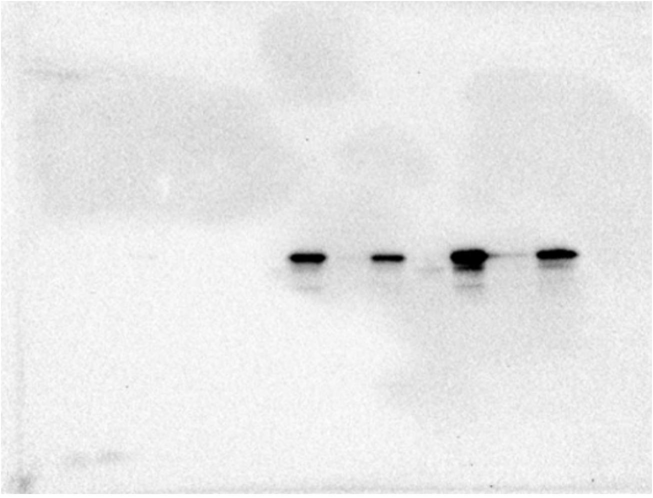

β-actin

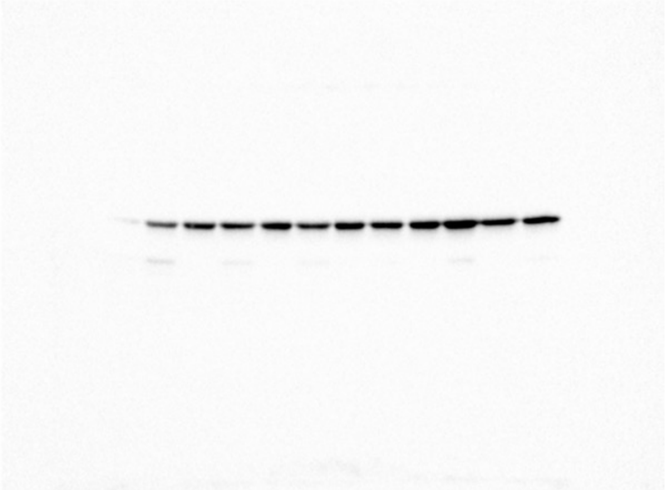

Figure S3C WB source data

Lysate anti-NLRP3

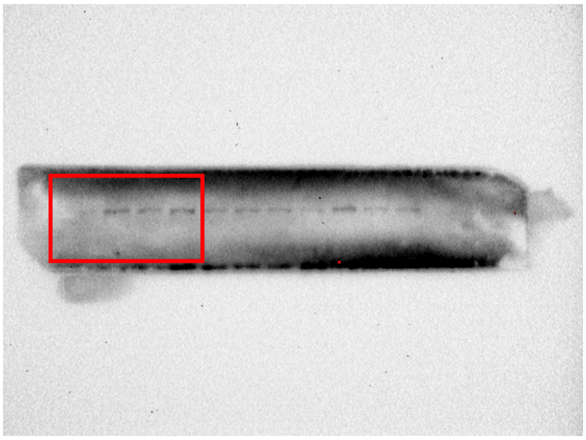

Lysate anti-IL-1β

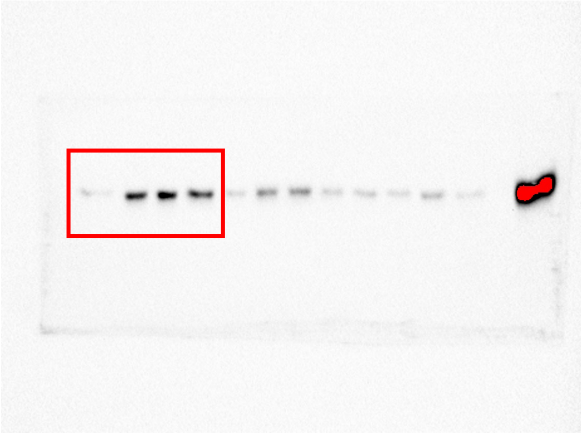

Lysate anti-β-actin (NLRP3)

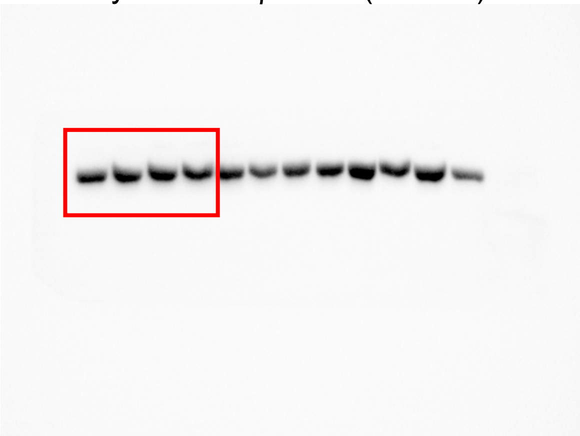

Lysate anti-β-actin (IL-1β)

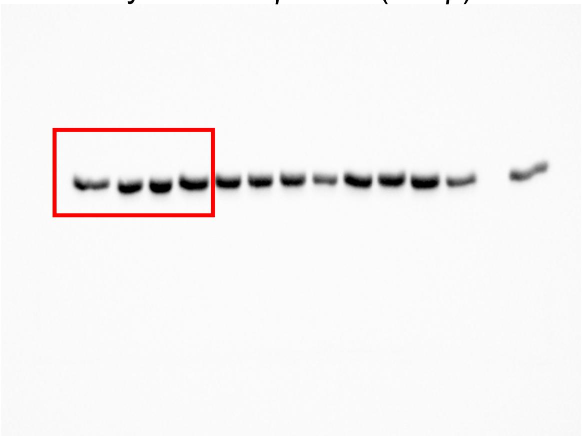

Supplement: Supplementary file 1 [file LSA-2023-02399_SdataF4_FS3.pdf]
